# Protein tyrosine kinase 6 regulates activation of SRC kinase

**DOI:** 10.1016/j.jbc.2022.102584

**Published:** 2022-10-10

**Authors:** Wanian M. Alwanian, Katarina Vlajic, Wenjun Bie, Andre Kajdacsy-Balla, Angela L. Tyner

**Affiliations:** 1 Department of Biochemistry and Molecular Genetics, University of Illinois at Chicago, Chicago, Illinois, USA; 2 Department of Pathology, University of Illinois at Chicago, Chicago, Illinois, USA; 3 University of Illinois Cancer Center, University of Illinois at Chicago, Chicago, Illinois, USA

**Keywords:** PTK6, SRC, BRK, tyrosine kinase, prostate cancer, erlotinib, CRPC, castration-resistant prostate cancer, FBS, fetal bovine serum, sc, single cell

## Abstract

Expression of Protein tyrosine kinase 6 (PTK6) is upregulated in several human solid tumors, and it has oncogenic roles in prostate and breast cancer. PTK6 and SRC kinase are distantly related, share many substrates, and often regulate the same signaling pathways, but whether they interact to regulate signaling is not well understood. We characterized crosstalk between PTK6 and SRC and show that PTK6 can directly phosphorylate SRC to promote its activation. Stable knockdown of PTK6 in multiple cancer cell lines leads to decreased activating phosphorylation of SRC. We show that coexpression of kinase-dead SRC and active PTK6 in mouse embryonic fibroblasts lacking *Src*, *Yes*, and *Fyn* results in activating phosphorylation of SRC. However, there is no reciprocal effect, because active SRC does not promote activating phosphorylation of PTK6. Overexpression of active PTK6 maintained activation of epidermal growth factor receptor (EGFR), AKT, and FAK, but not SHP2 and ERK1/2 in cells with knockdown of SRC. Both PTK6 and SRC are regulated by EGFR, and its inhibition with erlotinib downregulated PTK6 and to a lesser degree SRC activation in LNCaP cells that overexpress active PTK6. Erlotinib treatment also led to AKT inhibition, but overexpression of active PTK6 prevented this. Our data demonstrate overlapping and unique functions for PTK6 and SRC. Finally, we show that *PTK6* and *SRC* are coexpressed in subsets of human prostate and breast cancer cells, and active PTK6 and active SRC colocalize in prostate cancer, supporting a role for PTK6 in promoting SRC activity in cancer.

Protein tyrosine kinase 6 (PTK6), also called BRK, is part of an intracellular family of nonreceptor tyrosine kinases, which includes FRK and SRMS, and is distantly related to the SRC family of kinases. PTK6 harbors SH domains SH3, SH2, and tyrosine kinase SH1 domains, which are required for its function, but it lacks the N-terminal SH4 domain required for myristoylation/palmitoylation and membrane targeting (1). This results in flexibility in intracellular localization. PTK6 is activated by auto-phosphorylation on Tyr342 and inhibited by auto-phosphorylation on Tyr447, which engages the SH2 domain in an intramolecular interaction (2). PTK6 was first discovered in cultured human melanocytes (3), and then it was cloned and characterized in breast cancer and mouse intestinal epithelium (4, 5). Since then, it has been found to have context-dependent functions in different cancers, including prostate and breast cancer.

Targeting PTK6 to the plasma membrane promoted an epithelial mesenchymal transition and increased tumorigenesis of prostate tumor cells (6). In mouse models of breast and prostate cancer, disruption of *Ptk6* impairs tumorigenesis (7, 8). In these cancers, oncogenic functions of PTK6 have been associated with its activation at the plasma membrane (9, 10, 11). At the membrane, PTK6 interacts with and directly phosphorylates its substrates EGFR, AKT, focal adhesion kinase (FAK), BCAR1, and β-catenin (reviewed in (12). It can also promote activation of ERK1/2 (13). Loss of tumor suppressor PTEN, a frequent event in prostate cancer, contributes to PTK6 activation; PTEN dephosphorylates PTK6 on Tyr342 and inhibits its activity (8).

The nonreceptor tyrosine kinase SRC is the most studied member of the SRC family, and it shares around 44% homology with PTK6 family kinases (1). SRC is regulated by phosphorylation on Tyr416 (consistent with previous reports, we use chicken SRC numbering), located in the catalytic domain for activation and phosphorylation on Tyr527 (Tyr530 in human) within the C-terminal tail by the nonreceptor tyrosine kinase CSK for inhibition (14). When in an inactive conformation, the SH2 domain of SRC binds phosphorylated C-terminal Tyr527, while the SH3 domain interacts with the linker region between the SH2 and the kinase domain, leading to a closed inactive conformation (15). However, this interaction is suboptimal. Therefore, SRC can interact with many tyrosine phosphorylated proteins through its SH2 domain and proteins that harbor proline rich regions using the SH3 domain (16, 17). This suggests that intracellular interactions with SRC-binding partners can regulate activation of SRC kinase by competing with its domains engaged in the inhibition mechanism. SRC regulates tumorigenesis, including growth, survival, and metastasis, by integrating the cell surface with cytoplasmic signaling (18).

PTK6 and SRC couple signals to their downstream substrates, following the activation of different receptor tyrosine kinases. For instance, they can bind to the phosphorylated forms of EGFR and subsequently they are activated and sustain EGFR signaling by phosphorylation of EGFR on Tyr845 (19, 20). Furthermore, PTK6 and SRC activation have been correlated with resistance to HER2 inhibitors in breast cancer. In one study, PTK6 inhibition led to an increase in apoptosis of lapatinib-resistant HER2^+^ breast cancer cell lines (21), while inhibition of SRC kinase by SRC kinase inhibitors restored lapatinib sensitivity, in a different study (22).

Multiple studies indicate that targeting PTK6 and SRC may provide an advantage in cancer therapy although specificity of these inhibitors is lacking (23, 24). Different tyrosine kinase inhibitors have been developed that inhibit SRC kinase in clinical tumors, including dasatinib, which was developed as a dual SRC/ABL inhibitor. Although not specific, dasatinib has been shown to be effective as a SRC pathway inhibitor in multiple tumors (25). Interestingly, it has been shown in recent years that dasatinib can target and inhibit PTK6 in the nanomolar range (26, 27). As a result, it is plausible that the effect observed through dasatinib is mediated partially through PTK6 inhibition. Homology, similarities in regulatory mechanisms, and substrate recognition suggested that PTK6 and SRC might be involved in crosstalk and led us to investigate how these tyrosine kinases may interact. We show that PTK6 directly interacts with and phosphorylates SRC on Tyr416 and enhances its activity. This finding provides insight into the importance of PTK6 in cancer and the benefit of PTK6 inhibition in cancer therapy.

## Results

### PTK6 expression regulates SRC phosphorylation on Tyr416

**Figure 1:**
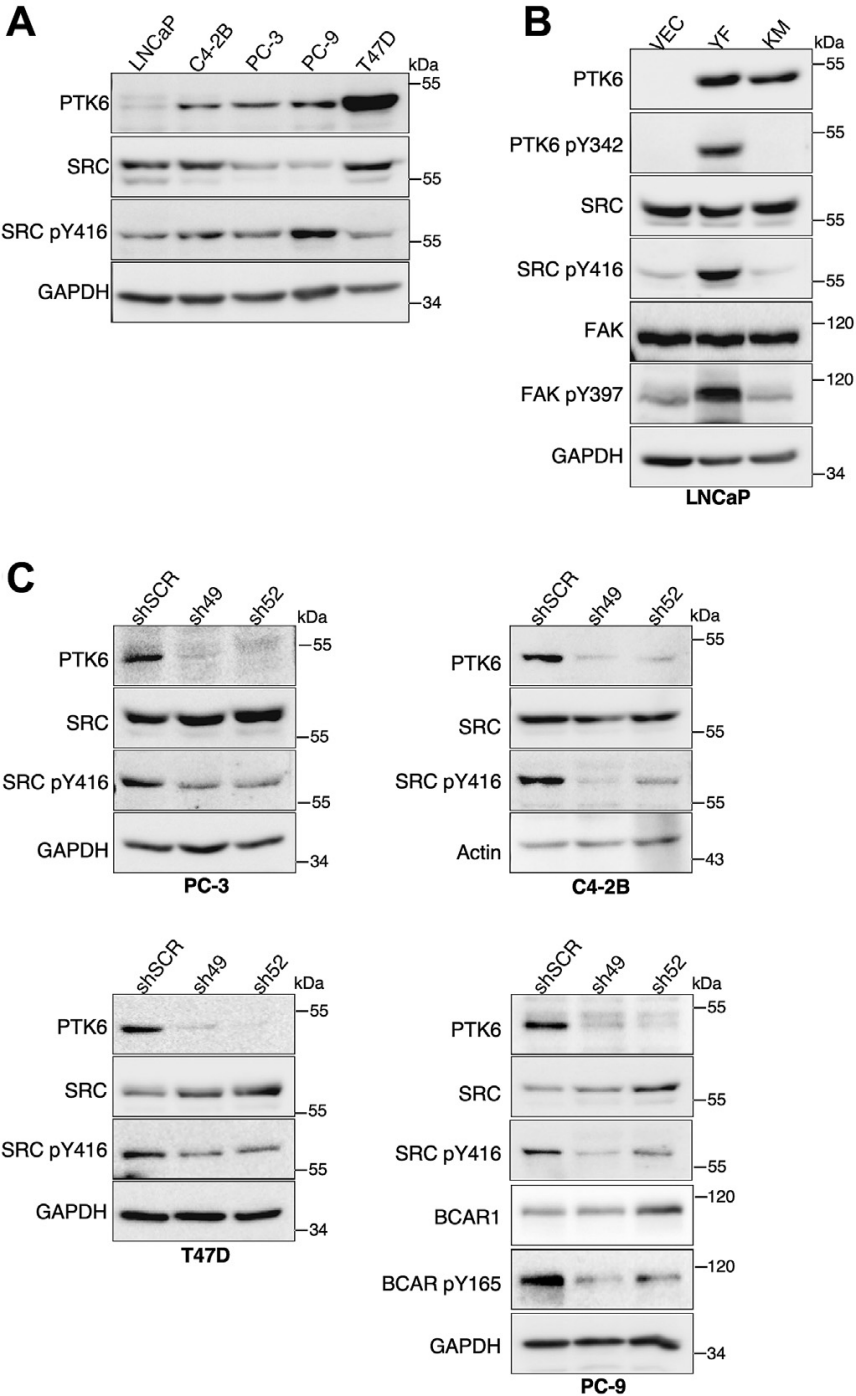
**PTK6 positively regulates SRC phosphorylation at Tyr416 in a kinase-dependent manner.***A*, differential expression of PTK6 and SRC in cancer cell lines of different origins, including prostate (LNCaP, C4-2B, and PC-3), lung (PC-9), and breast cancer (T47D). *B*, activating phosphorylation of SRC (pY416) was examined following transient transfection of empty vector (Vec), constitutively active PTK6-YF, or kinase-dead PTK6-KM in LNCaP cells. Total cell lysates were prepared for immunoblotting 24 h posttransfection. *C*, activating phosphorylation of SRC was examined following stable knockdown of PTK6 by two different shRNAs (sh49 and sh52) or scrambled control (shSCR) in PC-3, C4-2B, PC-9, or T47D cell lines. Total cell lysates were harvested and subjected to immunoblotting.

We examined PTK6 and SRC in three prostate cancer cell lines (LNCaP, C4-2B, and PC3) and in breast (T47D) and lung (PC-9) cancer cell lines (Fig. 1*A*). We chose several prostate cell lines, because the roles of PTK6 in prostate cancer have been extensively studied by our group, and we have shown that PTK6 regulates prostate cancer cell survival and metastasis (6) and prostate tumor formation *in vivo* (8). To explore if PTK6 might regulate SRC, we transfected empty vector and constructs expressing constitutively active PTK6 (YF), with a mutation of its regulatory C-terminal tyrosine (Tyr447) to phenylalanine or kinase-dead PTK6 (KM), bearing a mutation in the lysine 219 required for ATP binding into LNCaP cells, which express low levels of endogenous PTK6. A marker for SRC kinase activation is phosphorylation on tyrosine residue 416 (pY416) in the kinase domain, while active PTK6 is detected by phosphorylation of tyrosine 342 (pY342). We observed that active but not kinase-dead PTK6 induced activating phosphorylation of SRC (Fig. 1*B*).

Next, we looked at contributions of endogenous PTK6 to SRC activity in cell lines with higher levels of PTK6 expression. We silenced *PTK6* gene expression with two different well-validated shRNAs (28) in the PC-3 and C4-2B prostate cancer cell lines, the lung cancer cell line PC-9, and the breast cancer cell line T47-D. In all of these cell lines, we observed a decrease in SRC pY416 following knockdown of PTK6 (Fig. 1*C*), indicating that PTK6 is important for the activating phosphorylation of SRC. We also show reduced phosphorylation of BCAR1 (p130CAS), an established PTK6 substrate (11), following PTK6 knockdown in PC-9 cells.

### PTK6 directly phosphorylates SRC on Tyr416 and enhances its activity

**Figure 2:**
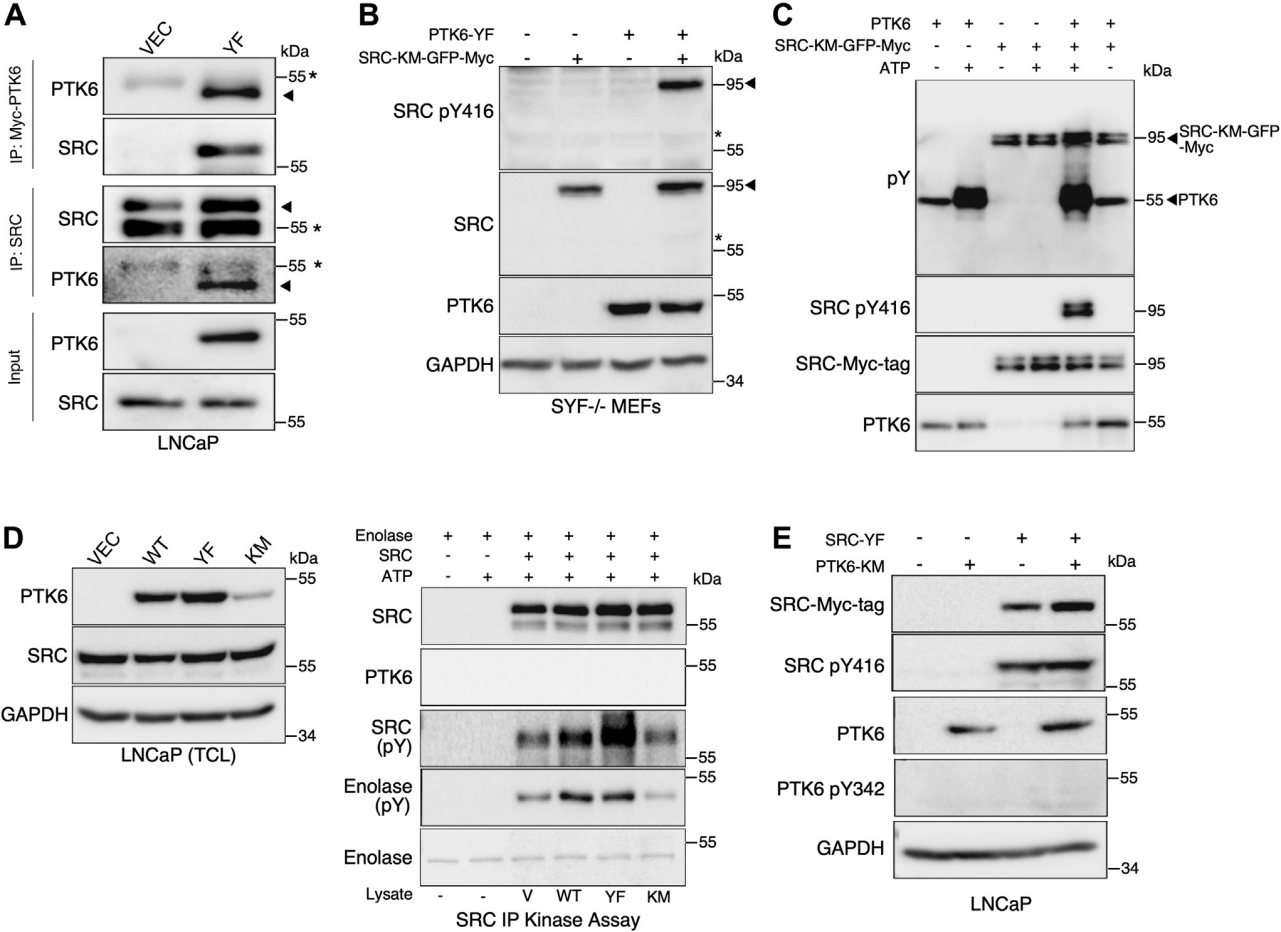
**PTK6 associates with and directly phosphorylates SRC at Tyr416.***A*, SRC and Myc-tagged PTK6 were immunoprecipitated from LNCaP cells that stably overexpress empty vector or constitutively active PTK6-YF. Protein complexes were subjected to immunoblot analysis. Arrowheads denote proteins of interest, while the asterisks at 55 KDa denote the background IgG heavy chain band. *B*, mouse embryonic fibroblasts deficient in *Src*, *Yes*, and *Fyn* (SYF) cells were transiently cotransfected with either empty vector (-) or constitutively active PTK6-YF or Myc/GFP-tagged kinase-dead SRC-KM. Cell lysates were harvested 24 h post transfection and subjected to immunoblotting. The asterisk denotes absence of any endogenous SRC or pTyr-416 in SYF cells. *C*, MYC/GFP-tagged kinase-dead SRC KM was overexpressed in SYF cells and immunoprecipitated using anti-Myc-tag, followed by incubation in kinase buffer with recombinant active human PTK6 plus or minus ATP for 10 min at 30 °C. Tyrosine phosphorylation of SRC and PTK6 was assayed with a mixture of PY20 and 4G10 phosphotyrosine antibodies. *D*, endogenous SRC was immunoprecipitated from LNCaP cells that stably overexpress empty vector (V), WT, constitutively active (YF), or kinase-dead (KM) PTK6 and assayed for activity by incubation with acid denatured rabbit muscle enolase and ATP for 10 min. Total cell lysates are shown in the left panel and the right panel shows the *in vitro* kinase assay. *E*, LNCaP cells that stably overexpress empty vector (-) or kinase-dead PTK6-KM were transiently transfected with empty vector or constitutively active SRC-YF, and activating phosphorylation of PTK6 (pY342) was examined by immunoblotting total cell lysates at 24 h post transfection.

SRC can be activated by engaging its binding partners and substrates to relieve the closed conformation and induce auto-phosphorylation (29). Since we observed that PTK6 expression regulates SRC Tyr416 phosphorylation, we investigated if PTK6 and SRC can interact in LNCaP cells. We transfected empty vector (control) or a construct expressing constitutively active PTK6-YF and immunoprecipitated PTK6 or endogenous SRC to examine complex formation within cells. SRC coimmunoprecipitated with PTK6, and PTK6 also coimmunoprecipitated with SRC (Fig. 2*A*).

To define the role of PTK6 more clearly, we ruled out the influence of other ubiquitous SRC-family tyrosine kinases, using SYF mouse embryonic fibroblasts that are null for *Src*, *Yes,* and *Fyn* tyrosine kinases. We coexpressed kinase-dead SRC (SRC-KM) that lacks the critical lysine residue required for ATP binding, with constitutively active PTK6-YF in SYF cells. When active PTK6 is expressed, we observed an increase in phosphorylation of SRC on Tyr416 suggesting that SRC can be phosphorylated on Tyr416 by PTK6 in the absence of SRC-family kinase activity (Fig. 2*B*).

To determine if PTK6 can directly phosphorylate SRC, we performed an *in vitro* kinase assay with purified recombinant PTK6 and immunoprecipitated Myc-tagged and GFP-tagged kinase-dead SRC (SRC-KM-GFP-MYC), which had been overexpressed in SYF cells. We showed that SRC-KM is not phosphorylated on Tyr416 in the absence of PTK6 in these cells (Fig. 2*B*). Immunoblotting with phospho-tyrosine antibodies or an antibody specific for Tyr416 phosphorylation shows that purified active PTK6 can directly phosphorylate SRC on Tyr416 (Fig. 2*C*). While antibodies detecting global tyrosine phosphorylation (pY) detect some background phosphorylation of SRC-KM-GFP-Myc, activating phosphorylation at tyrosine residue 416 (pY416) of SRC requires active PTK6 and ATP.

To examine if PTK6 regulates SRC activity, we performed *in vitro* kinase assays with immunoprecipitated endogenous WT SRC from LNCaP cells that overexpress vector, WT PTK6, constitutively active PTK6 (YF), or kinase-dead PTK6 (KM) (total cell lysates, Fig. 2*D*, left panel). Purified enolase was used as a substrate since it is commonly used to assess SRC activity. We observed that SRC is more active toward its substrate enolase when it is immunoprecipitated from cells overexpressing either WT or constitutively active PTK6, but not vector or kinase-dead PTK6 (Fig. 2*D*, right panel). Although enolase is also a substrate of PTK6, PTK6 protein was not detected in the assay (Fig. 2*D*, right panel). Collectively, these observations suggest that PTK6 can directly phosphorylate SRC on Tyr416 to enhance SRC activity.

As PTK6 can directly phosphorylate SRC at Tyr416, we wanted to investigate if there is reciprocal crosstalk where SRC might phosphorylate PTK6 at Tyr342. We overexpressed constitutively active SRC (SRC-YF) that has a mutation in the C-terminal inhibitory Tyr527 to phenylalanine so it cannot be inhibited, in LNCaP cells that stably overexpress kinase-dead PTK6 (PTK6-KM). We observed no increase in Tyr342 phosphorylation *in vitro*, indicating that SRC has no direct role in phosphorylation of PTK6 (Fig. 2*E*).

### PTK6 rescues AKT, FAK, and EGFR activation following SRC knockdown, but not SHP2-ERK1/2 activation

**Figure 3:**
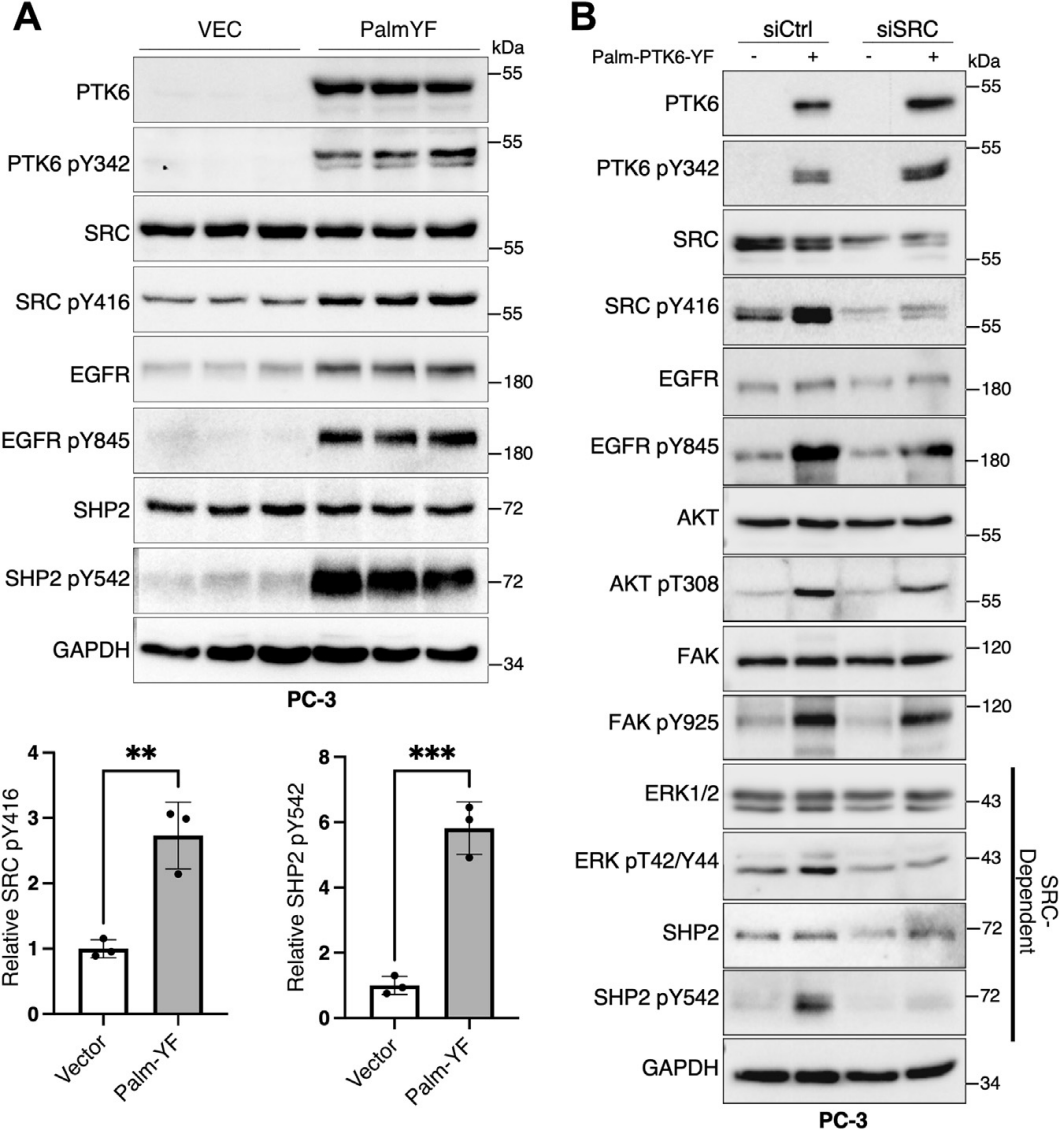
**SRC loss does not impact activation of AKT, FAK, and EGFR by PTK6.***A*, total cell lysates of PC-3 cells that stably express empty vector or membrane targeted-constitutively active PTK6 (Palm-YF) were harvested for immunoblotting (Top). Phospho-protein signals were quantified and normalized to total proteins, and two tailed Student's *t*-test was used for statistical analysis (Bottom), ∗∗*p* < 0.01, ∗∗∗*p* < 0.001. *B*, siRNA was used to knock down *SRC* gene expression in PC-3 cells stably expressing empty vector or membrane-targeted constitutively active PTK6 (Palm-YF). PC-3 cells were transfected with SRC siRNA (siSRC) or control siRNA (siCtrl) and harvested after 48 h. Total cell lysates were subjected to immunoblotting.

PTK6 activation at the plasma membrane has been reported in breast and prostate cancer (9, 10, 11). SRC promotes initiation of prostate cancer which is dependent on posttranslational palmitoylation and membrane localization (30). At the membrane, PTK6 can regulate signaling by EGFR, AKT, and ERK1/2 (13, 19, 31). The role of ERK1/2 activation by membrane-localized PTK6 has been thought previously to be mediated by EGFR and phosphorylation of FAK at the Grb2-binding site, Tyr925 (13, 32). Furcht *et al*. reported a mechanism by which SRC family kinases contribute to EGFR-mediated activation of the SH2 domain–containing protein phosphatase 2 (SHP2) (33). SHP2, a ubiquitously expressed tyrosine phosphatase, is activated by a variety of cytokine and growth factor initiated signaling pathways including PDGFR and EGFR and subsequently acts downstream of these pathways to stimulate signaling such as the RAS-MAPK, PI3K, and JAK-STAT pathways (34, 35, 36). SHP2 is activated by binding phosphorylated Gab1 (Grb2-associated binder protein 1) at Tyr627 and Tyr659 using the SH2-domain (37). SRC counteracts dephosphorylation of the adaptor protein GAB1, thereby protecting GAB1-SHP2 interaction and prolonging EGFR signaling (33). PTK6 activation of EGFR may contribute to this mechanism (19). To validate this, we used PC-3 cells that express empty vector or Palm-PTK6-YF and we observed upregulation of SRC activation at Tyr416 and SHP2 phosphorylation at Tyr542, an indication of its activation (Fig. 3*A*).

Numerous reports by our lab and others indicate that PTK6 shares substrates with SRC (reviewed in (12)). Hence, we sought to investigate if this activation can be partially regulated through SRC activation by PTK6. Using *SRC*-specific siRNA, we downregulated SRC expression in PC-3 cells that stably express empty vector or constitutively active-membrane–targeted PTK6 (Palm-YF). Ectopic expression of active PTK6 rescued activation of AKT, EGFR, and FAK following SRC knockdown (Fig. 3*B*). It has been demonstrated that PTK6 can directly phosphorylate these substrates to induce their activation (19, 31, 38). However, SRC expression and activation appear essential for PTK6-mediated activating phosphorylation of ERK1/2 and SHP2 (Fig. 3*B*), as ectopic expression of active PTK6 was not able to rescue loss of ERK1/2 and SHP2 activation after SRC knockdown, suggesting SRC is required to promote Ras-GTPase activity by directly phosphorylating RAS at Tyr32 (39).

### EGFR inhibition downregulates SRC, but not AKT activation by PTK6

EGFR is part of the ErbB family of receptor tyrosine kinases. Aberrant activation of EGFR is common in prostate tumors and correlates with shorter time of prognosis to castration-resistant prostate cancer (CRPC) (40). A study by Day et al. found that EGFR function in prostate cancer is important for primary and secondary sphere formation. Moreover, they observed that EGFR was expressed in metastatic circulating tumor cells (41). Using immunohistochemistry studies, Di Lorenzo *et al*. demonstrated that EGFR expression positively correlated with progression of prostate cancer to androgen independence (42). EGFR antagonists such as erlotinib, an EGFR inhibitor used in the treatment of lung and pancreatic cancers, are successful in inhibiting growth of these tumors even though resistance eventually develops (43).

**Figure 4:**
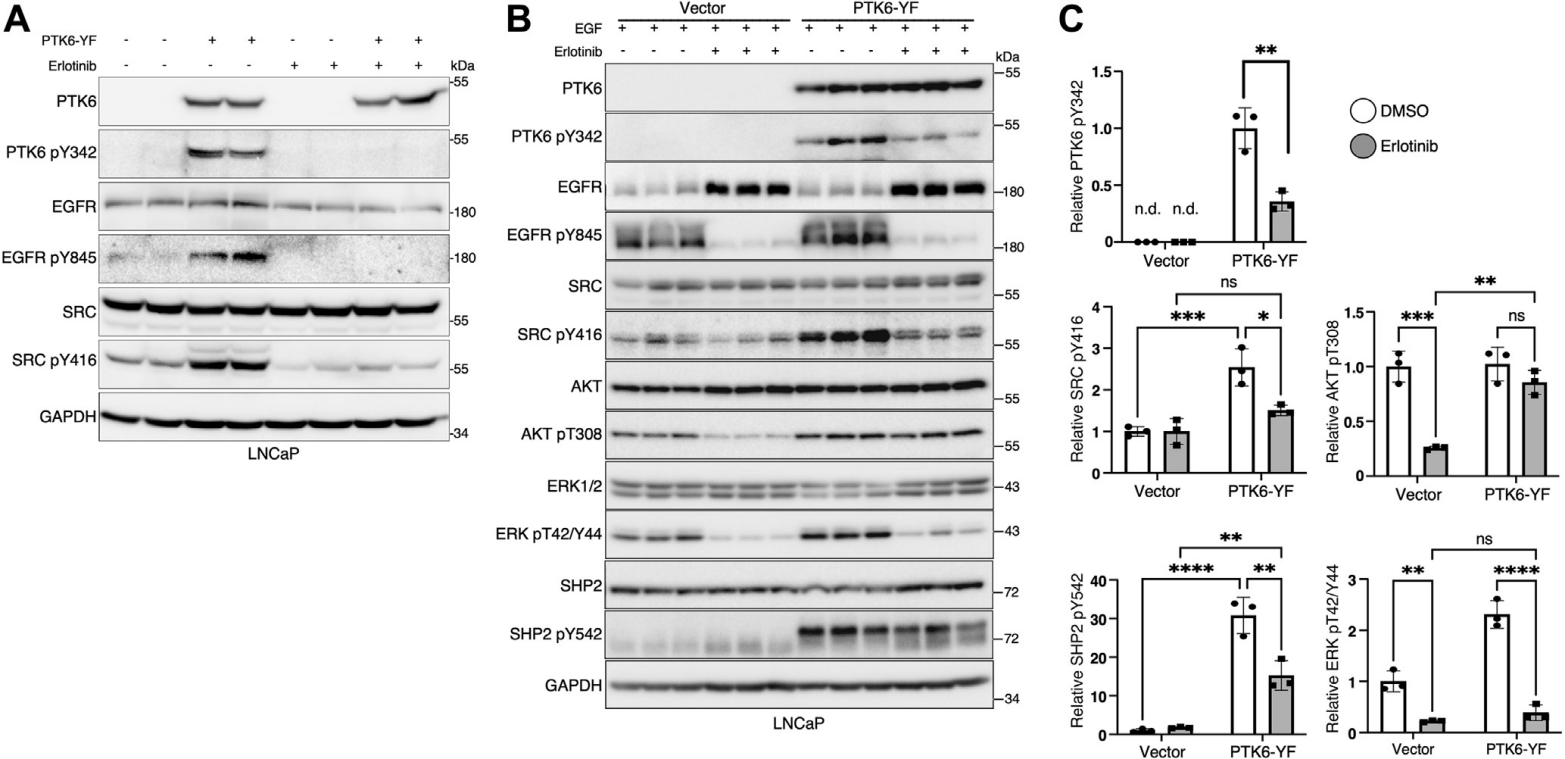
**EGFR inhibition impacts activation of SRC by PTK6, while AKT is rescued by PTK6 overexpression.***A*, erlotinib inhibits PTK6, SRC, and EGFR. LNCaP cells stably expressing empty vector or constitutively active PTK6-YF were treated with erlotinib for 1 h. *B*, erlotinib does not suppress PTK6 activation of AKT. Cells were starved overnight in triplicate, then treated with 1 μM erlotinib for 1 h. Before cells were harvested, they were treated with 50 ng/ml EGF for 10 min. Total cell lysates were subjected to immunoblotting to examine EGFR downstream signaling including PTK6, SRC, and their substrates. *C*, the relative effect of EGFR inhibition and PTK6 activation was normalized to total protein levels and quantified as illustrated for phospho-PTK6, phospho-SRC, phospho-AKT, phospho-ERK1/2, and phospho-SHP2. Statistical analysis was performed using two-tailed Student's *t-*test for phospho-PTK6 (pY342) and two-way Anova for the remaining phospho-proteins (n = 3, ∗*p* < 0.05, ∗∗*p* < 0.01, ∗∗∗*p* < 0.001, ∗∗∗∗*p* < 0.0001).

Both SRC and PTK6 cooperate with EGFR; they can be activated by the receptor and they can also further potentiate its activation (19, 20). The EGFR inhibitor inhibits EGFR, SRC, and PTK6 activity (Fig. 4*A*). To address the roles of PTK6 and SRC activation downstream of EGFR signaling, we used erlotinib to inhibit EGFR in epidermal growth factor (EGF)-stimulated LNCaP cells that stably express vector or constitutively active PTK6-YF (Fig. 4*B*). Expectedly, EGFR inhibition significantly downregulated PTK6 and ERK1/2 phosphorylation and activation. PTK6 was previously reported to bind and activate EGFR by phosphorylation of Y845 (19). Interestingly, EGFR inhibition did not impact basal SRC activity in LNCaP vector cells, but it significantly diminished SRC activation that was induced by active PTK6, although the levels of SRC activity still had upward trend in comparison to vector control cells (Fig. 4, *B* and *C*). Previously, we showed PTK6 activates AKT in response to EGF treatment in prostate cells (38). Here, we show that erlotinib-mediated EGFR inhibition reduced basal AKT activation in vector cells, but it had no impact on AKT activation in cells that overexpress PTK6-YF, suggesting that under these conditions, PTK6 plays an important role in AKT activation despite EGFR or SRC inhibition (Fig. 4, *B* and *C*). We also show that active PTK6 significantly induced activation of SHP2 even though EGFR activity was downregulated with erlotinib (Fig. 4, *B* and *C*).

### *PTK6* and *SRC* are coexpressed in a subset of cancer cells

**Figure 5:**
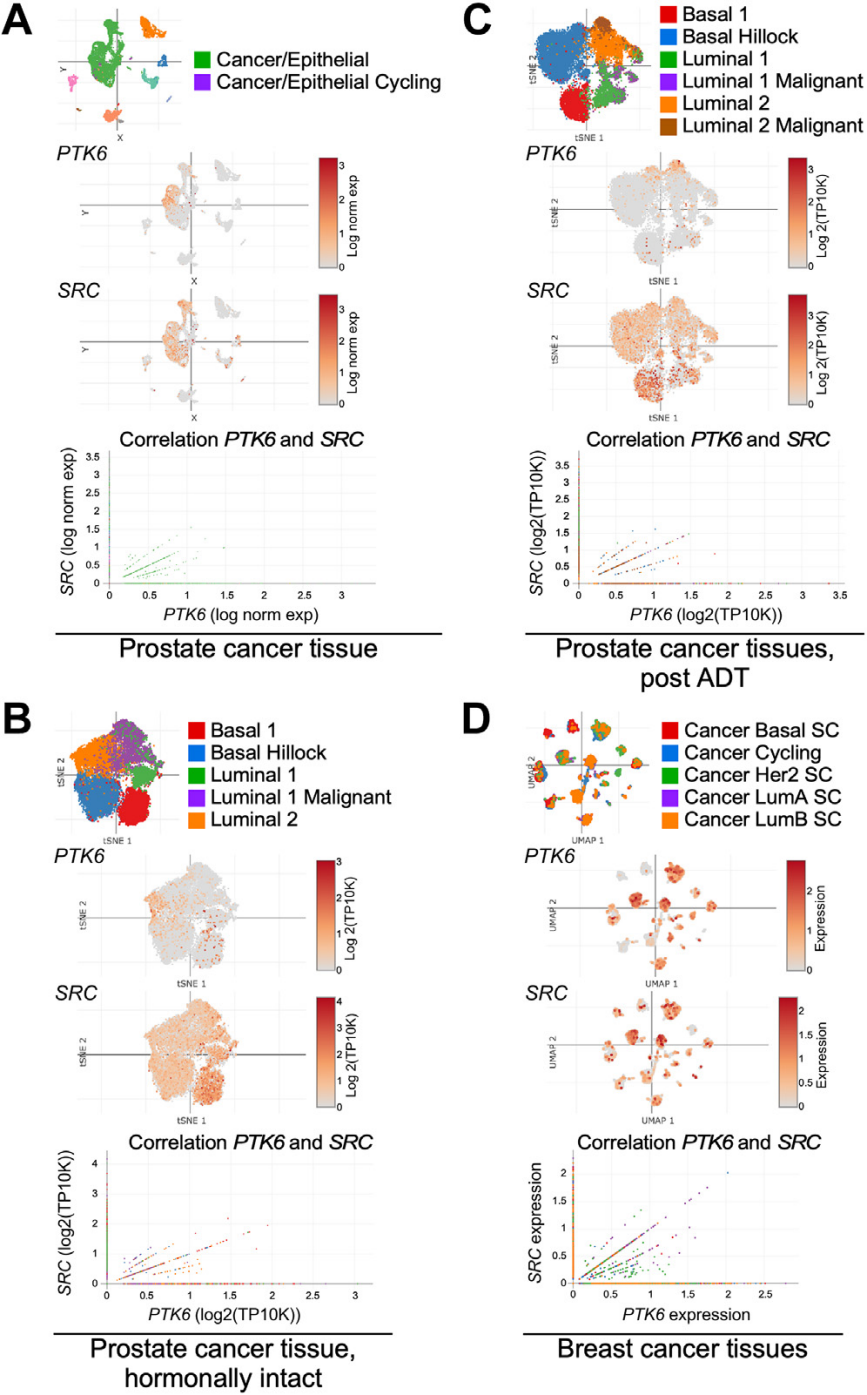
**Analysis of scRNA-seq data from The Broad Institute Single Cell Portal shows coexpression of *PTK6* and *SRC* in the same cells in prostate and breast tissues.** Cell type–specific scatter plots show epithelial cell populations alone or with different immune and endothelial cells. Only colors of epithelial cell populations are labeled for simplicity. Expression of *PTK6* and *SRC* are represented as scatter plots, corresponding to the cell type–specific plots. Plots showing correlation between *PTK6* and *SRC* contain all cell population data (not only epithelial cells). *PTK6* and *SRC* expression positively correlate in cells that express both genes. *A*, primary prostate cancer tissue (45), emphasizing only epithelial cancer cells. *PTK6* is mostly present in a subset of cancer/epithelial cells, while *SRC* is present throughout epithelial cancer and in other cell populations. *B*, *PTK6* and *SRC* are expressed in epithelial cells from prostate cancer tissues derived from hormonally intact patients, depleted for cells having copy number alterations (CNAs) (44) and (*C*) epithelial cells from prostate cancer tissues derived from patients that underwent androgen deprivation therapy (ADT), depleted for cells having CNAs (44). Elimination of cells with CNA led to elimination of the most of malignant cells. *PTK6* mRNA expression is the highest in malignant and luminal 2 cell populations, while *SRC* is present in all cell types. *D*, *PTK6* and *SRC* are coexpressed in epithelial cancer cells from primary breast cancer tissues (46). *PTK6* mRNA is present in all cancer cell types, being a bit higher in LumA SC and Her2 SC. SC represents the annotation for cell populations that are generated using a bioinformatic single-cell method of intrinsic subtype classification developed by Wu et al. (45).

SRC is widely expressed in multiple cell types and tissues, while PTK6 exhibits a more restricted pattern of expression and has been studied primarily in epithelia and solid tumors. To determine the possible biological significance of PTK6 regulation of SRC, we examined publicly available single cell (sc) RNA-seq datasets available on The Broad Institute Single Cell Portal. Because of its established roles in prostate and breast cancer, we explored how *PTK6* and *SRC* are coexpressed in nonmalignant and malignant prostate (44, 45) (Fig. 5, *A*–*C*) and breast cancer tissues (46) (Fig. 5*D*). While *SRC* mRNA expression is more widely distributed, distinct subsets of cells coexpress both *PTK6* and *SRC* in both cancers. In the prostate cancer, *PTK6* is expressed only in a portion of cancer epithelial cells (Fig. 5*A*, green and purple cell populations), while *SRC* is more broadly distributed in epithelial cells and other cell populations, such as immune cells (pink, light-orange), fibroblasts (blue), perivascular (light-green), and endothelial cells (orange) (Fig. 5*A*, other cell populations colors not labeled) (45). There is a positive correlation between *PTK6* and *SRC* expression in cells where they are both expressed. In the Karthaus *et al* dataset (44), which analyzed epithelial cells that are depleted for cells with copy-number alterations, *PTK6* is localized primarily to the luminal 2 population (Fig. 5*B*) and malignant luminal 2 group, representing cells without copy-number alterations but still classified as cancer cells (Fig. 5*C*). In comparison, *SRC* has more widespread expression in all epithelial populations. The analysis of the breast cancer scRNAseq dataset shows that *PTK6* and *SRC* are highly expressed in cells from all cancer types (basal, cycling, HER2+, Luminal A, and Luminal B) (Fig. 5*D*) (46) with a high correlation between *PTK6* and *SRC* expression in these cells.

**Figure 6:**
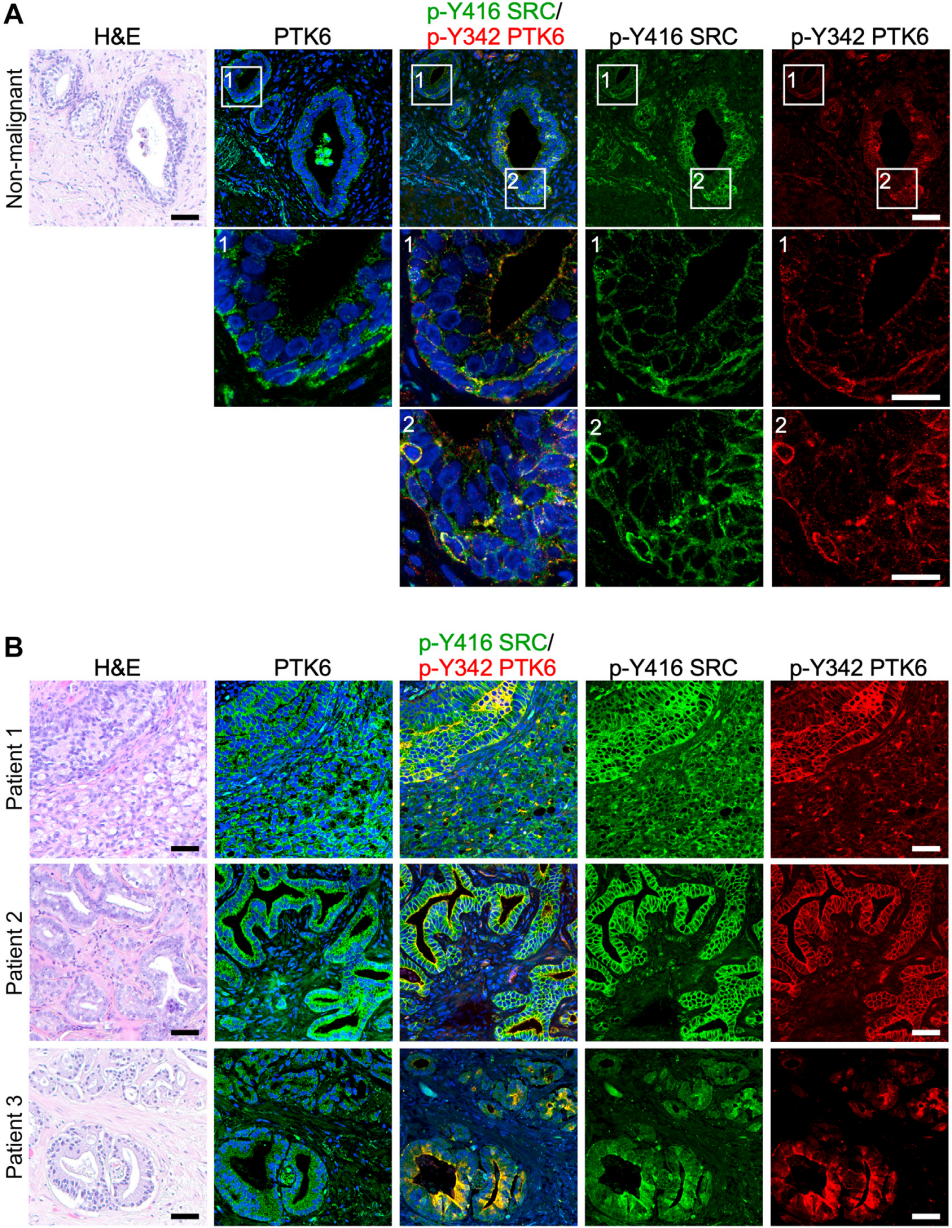
**Active PTK6 and active SRC colocalize in CRPC tissues.** Serial sections of CRPC were stained for total PTK6, or double stained for pY342 (active PTK6), and pY416 (active SRC) and stained with hematoxylin and eosin (H&E) to show morphology. *A*, nonmalignant region of a human prostate tumor, showing localization of total PTK6, active PTK6, and active SRC. Active PTK6 and active SRC are found colocalized in the same cells. Higher magnifications of the boxed regions 1 and 2 are shown. Scale bar: upper panel 50 μm, lower panels 20 μm. *B*, malignant tissues from three different CRPC patients showing localization of PTK6, active PTK6, and active SRC in the same regions of the tumors. Active PTK6 and active SRC are localized in the same cells in cancer regions. The scale bars represent 50 μm. CRPC, castration-resistant prostate cancer.

To explore PTK6-SRC crosstalk in prostate cancer, we examined colocalization of active PTK6 with active SRC in samples of transurethral resection of the prostate from patients who were diagnosed with CRPC. This procedure is used to relieve symptoms of prostate cancer such as urinary dysfunction (47). Using immunofluorescence, we double-stained for PTK6 and SRC kinase activation, pY342, and pY416, respectively, in sections of nonmalignant prostate and CRPC. Serial sections were also stained for total PTK6 and with H & E and scored by a pathologist. In the nonmalignant prostate tissue (Fig. 6*A*), total PTK6, active PTK6, and active SRC are localized to the glands, with highest levels found at the basal side of gland, possibly the basal cells. Cancer tissues from three different patients exhibit higher expression of PTK6 and stronger activation of both PTK6 and SRC (Fig. 6*B*) in comparison to the nonmalignant tissue (Fig. 6*A*). In prostate cancer (Fig. 6*B*), active PTK6 and active SRC colocalize within the same cells. Colocalization of active PTK6 and active SRC in tissues indicates possible crosstalk and a role for PTK6 in SRC activation *in vivo*.

## Discussion

Increased expression and amplification of *PTK6* have been reported in multiple tumor types, including breast, prostate, ovarian, skin, lung, and colon cancer, and a growing body of evidence indicates that PTK6 plays important roles in promoting tumorigenesis (reviewed in (23). While expression of SRC is ubiquitous, PTK6 expression is more restricted and primarily expressed in the linings of the gastrointestinal tract (5) and skin (48), with lower levels expressed in normal prostate (9) and breast tissue (10). However, PTK6 is overexpressed in breast cancer and prostate cancer (49, 50), and it is activated at the plasma membrane in these tumors (6, 10). Several interacting proteins and substrates of PTK6 have been identified including beta-catenin (28), AKT (38), FAK (31), BCAR1 (11), and EGFR (19). Interestingly, these signaling proteins are also SRC substrates (51, 52, 53, 54).

**Figure 7:**
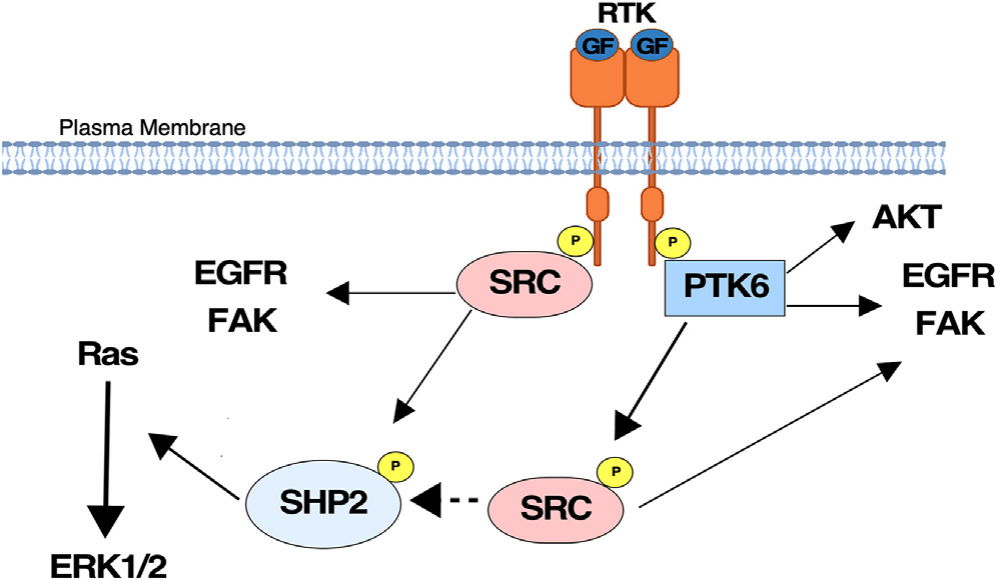
**PTK6 may enhance SRC signaling downstream of receptor tyrosine kinases.** A schematic showing roles of PTK6 and SRC in receptor tyrosine kinase signaling is illustrated. Activation of receptor tyrosine kinases results in recruitment and activation of PTK6 and SRC. PTK6 activates SRC, AKT, FAK, EGFR, SHP2, and ERK1/2. SRC activity appears to be important for the ability of PTK6 to activate the RAS-MAPK pathway through SHP2 activation. Although PTK6 activates AKT independent of SRC or EGFR activity, the activation of EGFR and FAK and possibly other substrates may be promoted by PTK6 through SRC activation.

Historically, SRC was thought to be activated by autophosphorylation. In 1985, Courtneidge discovered that SRC requires dephosphorylation to be activated (55). This suggests that SRC normally exists in an inactive conformation. Later, analysis of crystal structure of SRC resolved steps in SRC activation which involves opening of the kinase and autophosphorylation (56). Here, we describe a mechanism by which PTK6 can influence SRC signaling (Fig. 7). We show that ectopic active PTK6 enhances activating phosphorylation of SRC at Tyr416, and knockdown of endogenous PTK6 results in downregulation of SRC Tyr416 phosphorylation in different cancer cell lines.

SRC binding partners may promote its activation by competing with the inhibitory mechanism that involves intramolecular interactions between SH2 domain and the phosphorylated C-terminal Tyr527 by CSK. We find that kinase-dead PTK6 cannot activate SRC, suggesting that the PTK6 SH2 or SH3 domains alone are not sufficient for SRC activation. Only expression of constitutively active PTK6 caused increased SRC Tyr416 phosphorylation. Our data show that kinase-dead SRC can be phosphorylated on Tyr416 in the absence of major SRC family kinases, when active PTK6 is present (Fig. 2*B*). In addition, using phospho-tyrosine–specific antibodies and *in vitro* kinase assays, we obtained evidence that PTK6 directly phosphorylates SRC on Tyr416. However, this mechanism appears to be unidirectional since we did not observe SRC regulation of PTK6 phosphorylation on Tyr342. PTK6 and SRC both act downstream of receptor tyrosine kinases and contribute to the robustness of their signaling (57). It is possible that SRC, through activating growth factor receptors, promotes PTK6 activation, thereby enhancing its own activation. Synergy between PTK6 and SRC may be important in promoting tumorigenesis.

SHP2 is a phosphatase that activates the SRC and Ras pathways following growth factor receptor activation (58, 59, 60). Previous work suggested that membrane-targeted active PTK6 activates the MAPK pathway, but the mechanism had not been elucidated (13). Here, we show that through promoting activation of SRC, PTK6 activates SHP2 and subsequently, ERK1/2. Our finding that PTK6 is unable to rescue SHP2 and ERK1/2 activation when *SRC* expression is downregulated suggests a critical role for SRC in promoting SHP2 and ERK1/2 activation. However, both PTK6 and SRC directly target other substrates such as FAK and EGFR (Fig. 7).

Multiple reports indicate that EGFR is upstream of PTK6 and SRC (19, 20, 33). As a result, it is likely that aberrant activation and expression of EGFR influences both SRC and PTK6 activity, promoting their roles in tumorigenesis. We show that erlotinib, an FDA-approved EGFR inhibitor for the treatment of lung cancer (61) inhibits PTK6, AKT, and ERK1/2 in cells that overexpress active PTK6. Erlotinib-mediated EGFR inhibition also downregulated the robust activation of SRC and ERK1/2 observed in the presence of active PTK6. However, AKT activation, and to a lesser degree, SHP2 activation was not affected (Fig. 4*B*). This indicates that PTK6 can have an independent role in activating different pathways despite EGFR or SRC inhibition. For example, PTK6 has also been shown to promote activation of the IGF-1 receptor, which could lead to the sustained activation of AKT (62).

In prostate cancer, PTK6 activity negatively correlates with PTEN expression (8, 31). PTEN, a tumor suppressor, negatively regulates PTK6 activity by dephosphorylating PTK6 on Tyr342. Activation of SRC has been shown to reduce PTEN activity (63). This suggests that PTK6, through activation of SRC, may sustain activation of AKT and PI3K, as well as enhance its own activity. PTK6 and SRC are active and colocalize to the same regions in CRPC. This synergic act suggests that targeting these kinases may provide therapeutic potential in cancer treatment.

While there are currently no specific inhibitors for PTK6, several inhibitors inhibit PTK6 through an off-target effects (13, 23, 27). Dasatinib, a dual SRC/ABL kinase inhibitor, has been shown to inhibit PTK6 in the nanomolar range (26, 27). In a study conducted by Jiang *et al*., a small molecule inhibitor called XMU-MP2 inhibited PTK6 and resulted in breast cancer growth inhibition *in vitro* and *in vivo*. However, their screen showed that this inhibitor targets multiple tyrosine kinases, including EGFR, with different IC50 values (64). Vemurafenib, an inhibitor of mutant BRAF used for the treatment of melanoma, also inhibits PTK6, but not SRC, at nanomolar concentrations (65, 66). Vemurafenib inhibited PTK6 activity and oncogenic signaling in prostate cell lines, as well as xenograft prostate tumor growth (13). While a variety of data suggest SRC would be a good therapeutic target in cancer, SRC inhibitors were not effective when used alone (67). Since PTK6 and SRC target many of the same substrates and PTK6 can promote SRC activation, drugs that target both kinases may have greater efficacy than targeting either kinase alone in cancer therapy.

## Experimental procedures

### Cell culture and reagents

LNCaP (CRL-1740), C4-2B (CRL-3315), T-47D (HTB-133), HEK293 (CRL-1573), and Phoenix-AMPHO (CRL-3213) cells, and SYF (CRL-2459) mouse embryonic fibroblasts were obtained from ATCC. PC-9 cells were obtained from Sigma. LNCaP and C4-2B cells were cultured in RPMI with 10% fetal bovine serum (FBS). PC-3 cells were cultured in F12K with 10% FBS. PC-9 cells were cultured in RPMI with 5% FBS. Human embryonic kidney 293 cells and SYF cells were cultured in Dulbecco’s modified Eagle’s medium (DMEM) with 10% FBS. EGF from mouse was purchased from Sigma (E5160). For EGFR inhibition, erlotinib (Thermo Fisher Scientific) is used at 1 μM for 1 h.

### Protein lysates, immunoprecipitation, and immunoblotting

Preparation of total cell lysates and immunoprecipitation experiments were previously described (28). Proteins were probed with the following primary antibodies: PTK6 (1:500 for IB, 1:100 for IF; Santa Cruz Biotechnology, sc-166171), active PTK6-pTyr342 (1:1000 for IB, 1:100 for IF; Millipore, 09-144), SRC (1:1000; Cell Signaling, 36D10), mouse monoclonal SRC (1:1000, Millipore, GD11), active SRC-pTyr416 (1:1000 for IB, 1:100 for IF; Cell Signaling, 2101), FAK (1:1000; Santa Cruz Biotechnology, sc-558), active FAK-pTyr397(1:1000; Cell Signaling, 8556), active FAK-pTyr925 (1:1000; Cell Signaling, 3284), BCAR1 (1:1000; BD Biosciences 610271), BCAR1 p-Y165 (1:1000; Cell Signaling 4015), Myc-tag (1:1000, Cell Signaling, 2276), Phosphotyrosine PY20 (1:5000; Santa Cruz, sc-508), Phosphotyrosine 4G10 (1:5000, Millipore, 05-321), EGFR (1:1000; Cell Signaling, 2232), active EGFR-pTyr845(1:1000; Cell Signaling, 2231), AKT (1:1000; Cell Signaling, 9272), Active AKT-pThr308(1:1000; Cell Signaling, 9275), ERK1/2 (1:1000; Cell Signaling, 9102), active ERK1/2-pThr42/Tyr44 (1:1000; Cell Signaling, 9106), SHP-2 (1:1000; Cell Signaling, 3752), active SHP-2-pTyr542(1:1000; Cell Signaling, 3751), GAPDH (1:1000; Cell Signaling, 2118), and β-actin (1:1000; Sigma, A5441).

### Constructs, transfections, RNA interference, and lentiviral infections

pcDNA3 vector containing PTK6-WT, PTK6-YF (active), and PTK6-KM (dead mutant) was previously described (28). pUse vector containing a MYC epitope–tagged human SRC-YF (active) and MYC epitope– and GFP-tagged human SRC-KM (inactive) (68) were kindly provided by Dr Andrei Karginov (University of Illinois at Chicago). For transient transfections, LNCaP cells were plated on 10 cm plates in media lacking antibiotics and were transfected the following day with 8 μg of empty vector or PTK6 expression constructs using lipofectamine 2000 for 24 h. Similarly, for SRC-YF expression, LNCaP cells overexpressing empty vector or PTK6-KM were transfected with SRC-YF construct. SYF cells were cotransfected with 4 μg of empty vector, PTK6-YF or SRC-KM alone, or both constructs together for 24 h. Dicer substrate siRNAs were purchased from Integrated DNA Technologies. The sequence for control is (5′-CGUUAAUCGCGUAUAAUACGCGUAT-3′). The sequence for Dsi-SRC is (5′-CCCUUCGAGAUCAUCACUUCCUUGC-3′). For RNA interference gene silencing, 2 × 10^5^ PC-3 cells stably expressing empty vector control or palm-PTK6-YF were plated in 6-well plate and transfected the following day with 150 nM of siCtrl or siSRC using lipofectamine 2000. Transfection media was changed 24 h and cells were harvested 48 h posttransfection. For Lentiviral infections, pBabe-Vector, pBabe-PTK6-WT, pBabe-PTK6-YF, or pBabe-PTK6-KM were transfected in Phoenix-AMPHO cells using lipofectamine 2000 and DMEM with 10% FBS, and viruses were collected 48 h and 72 h posttransfection and used to infect LNCaP cells which was selected with 2 μg puromycin.

### *In vitro* kinase assay

SYF cells were transfected with SRC-KM-GFP-Myc plasmid. Twenty four hours posttransfection, cells were lysed with RIPA buffer (20 mM Tris–HCL, PH 7.5, 150 mM NaCl, 1 mM Na_2_ EDTA, 1 mM EGTA, 1% NP-40, 1% sodium deoxycholate, 2.5 mM sodium pyrophosphate, 1 mM ß-glycerophosphate, and 1 mM Na_3_Vo_4_), and SRC-KM-GFP-MYC was immunoprecipitated with Myc-tag antibody. Fifty nanograms of purified recombinant PTK6 (Millipore, MA) was incubated with immunoprecipitated SRC-KM in 30 μl kinase buffer (20 mM Hepes, 150 mM NaCl, 1 mM DTT, 10 mM MnCl_2_, and 0.01% Triton X-100) for 10 min at 30 °C. The reaction was terminated by adding 30 μl of 2× laemmli sample buffer and boiling.

### Human tissue samples

The samples from CRPC patients were identified by pathologist Dr Andre Kajdacsy-Balla (University of Illinois at Chicago) and their use approved by The University of Illinois at Chicago Institutional Review Board (IRB). Areas showing staining for PTK6, pY342 PTK6, and pY416 SRC in adjacent tissue sections were compared to H&E staining.

### Immunofluorescence

Paraffin-embedded CRPC tissue sections were deparaffinized using xylenes and dehydrated using ethanol prior to antigen retrieval. For antigen retrieval, slides with tissue sections were incubated in 0.01 M citrate buffer (pH 6.0) at 95 °C (sub-boiling temperature) for 20 min. Tissue sections were blocked in 3% bovine serum albumin with 1% goat or horse serum in TNT buffer (0.1 M Tris–HCl pH 7.5, 150 mM NaCl, 0.05% Tween 20) for 1 h, at room temperature. Incubation with first primary antibodies (active SRC pTyr416 and total PTK6) was done overnight at 4 °C. Incubation with first secondary biotinylated anti-rabbit IgG antibodies made in goat (1:200 dilution; Vector Laboratories Inc, BA-1000) or anti-mouse IgG antibodies made in horse (1:200 dilution; Vector Laboratories Inc, BA-2000) was done for 1 h, at room temperature, followed by incubation with streptavidin-Alexa Fluor 488 conjugate (Invitrogen, Thermo Fisher Scientific, S32354) for 30 min. For double staining, slides stained for active SRC were blocked overnight with AffiniPure Fab Fragment Goat Anti-rabbit IgG (1:30 dilution; Jackson ImmunoResearch, 111-007-003) at 4 °C. Incubation with second primary antibody (active PTK6 pTyr342) was done overnight at 4 °C. Incubation with secondary anti-rabbit IgG Alexa Fluor A594 conjugate (1:100 dilution; Cell Signaling Technology, 8889) was done for 10 min at room temperature. Nuclei were stained with 4′,6-diamidino-2-phenylindole for 5 min.

### Publicly available data analysis

Data for single cell expression of *PTK6* and *SRC* mRNAs and their correlation were obtained from and visualized using The Broad Institute Single Cell Database Portal (https://singlecell.broadinstitute.org/single_cell). scRNA-seq datasets for prostate cancer (44, 45) and breast cancer (46) were interrogated.

### Statistics

Immunoblot quantification was performed using image studio lite software (LI-COR Biosciences). Statistical analyses were performed using GraphPad Prism software version 9.2 (GraphPad software) with at least three independent experiments. Two tailed Student's *t*-test was used to analyze one variable experiments. Two-way Anova was used for analysis of combinations of two categorical variables. The results are shown as the mean ± SD.

## Data availability

The manuscript contains all data described within the text.

## Conflict of interests

The authors have no conflicts or competing interests to report.
